# The Airway Epithelium—A Central Player in Asthma Pathogenesis

**DOI:** 10.3390/ijms21238907

**Published:** 2020-11-24

**Authors:** Jenny Calvén, Elisabeth Ax, Madeleine Rådinger

**Affiliations:** 1 Krefting Research Centre, Institute of Medicine at the Sahlgrenska Academy, University of Gothenburg, 405 30 Gothenburg, Sweden; jenny.calven@gu.se (J.C.); elisabeth.ax@astrazeneca.com (E.A.); 2 Translational Science and Experimental Medicine, Research and Early Development, Respiratory & Immunology, BioPharmaceuticals R&D, AstraZeneca, 431 83 Gothenburg, Sweden

**Keywords:** airway epithelium, asthma, barrier, allergen, virus, alarmins, type 2 inflammation, miRNA

## Abstract

Asthma is a chronic inflammatory airway disease characterized by variable airflow obstruction in response to a wide range of exogenous stimuli. The airway epithelium is the first line of defense and plays an important role in initiating host defense and controlling immune responses. Indeed, increasing evidence indicates a range of abnormalities in various aspects of epithelial barrier function in asthma. A central part of this impairment is a disruption of the airway epithelial layer, allowing inhaled substances to pass more easily into the submucosa where they may interact with immune cells. Furthermore, many of the identified susceptibility genes for asthma are expressed in the airway epithelium. This review focuses on the biology of the airway epithelium in health and its pathobiology in asthma. We will specifically discuss external triggers such as allergens, viruses and alarmins and the effect of type 2 inflammatory responses on airway epithelial function in asthma. We will also discuss epigenetic mechanisms responding to external stimuli on the level of transcriptional and posttranscriptional regulation of gene expression, as well the airway epithelium as a potential treatment target in asthma.

## 1. Introduction

Affecting more than 300 children and adults worldwide [1], asthma is a chronic inflammatory disease characterized by chest tightness, variable airflow limitation, coughing, wheezing and airway hyperresponsiveness to environmental triggers (i.e., allergens, pollen, animal dander, tobacco smoke and air pollution) [2,3]. Asthma symptoms are a result of an ongoing chronic airway inflammation. Allergic asthma is the most common type, where reversible airway limitation is caused by allergic airway inflammation and allergic sensitization is the major risk factor. Allergen exposure in sensitized individuals typically triggers a type 2 (T2)-biased inflammatory response. In the sensitization phase, inhaled allergens are captured by dendritic cells (DCs) and presented to naive CD4^+^ T cells in the presence of coactivators, including epithelial-derived cytokines, which promotes activation and polarization of T helper 2 (Th2) cells that produce IL-4, IL-5, and IL-13 [3,4]. These T2 cytokines are also produced by type 2 innate lymphoid cells (ILC2s) and are prominent orchestrators of the allergic inflammatory cascade that occurs in asthma. IL-4 drives isotype switching of B cells and production of IgE, which binds to the high affinity IgE receptor on mast cells. Allergen re-exposure results in allergen-mediated IgE cross-linking, which causes rapid mast cell activation and degranulation. IL-5 promotes airway eosinophilia, IL-4 and IL-13 act directly on the airway epithelium to induce goblet cell metaplasia and mucus hypersecretion, and IL-13 mediates airway hyperresponsiveness by effects on airway smooth muscle cells [4].

While allergic and non-allergic asthma are the most common asthma phenotypes, these can be further divided into a variety of subgroups, including eosinophilic or non-eosinophilic asthma, as well as late-onset asthma [5]. Adding to the complexity, asthma phenotypes are driven by different immunological mechanisms, so-called endotypes. Thus, to date it is clear that asthma is a heterogeneous disease [6,7]. However, current knowledge of the underlying molecular mechanisms in asthma subgroups is limited, and more information is needed in order to improve disease diagnosis and treatment regimes.

The airway epithelium is the first line of defense against pathogenic environmental factors such as allergens, pollution, viruses, fungi, and bacterial infections [8]. Hence, the airway epithelium plays an important role in initiating host defense and controlling immune responses and plays a key role in disease development and progression in asthma [8,9].

## 2. The Structure and Function of the Airway Epithelium

All surfaces of the mammalian body are covered with epithelial cells, including the skin, the gastrointestinal tract, and the airways from the nose and mouth all the way down to the alveoli. Though the structure and functions of epithelial cells differ depending on their location, they all are tightly interconnected through epithelial junctions. This reveals one key role of the epithelium in serving as a physical barrier against the environment.

**Figure 1:**
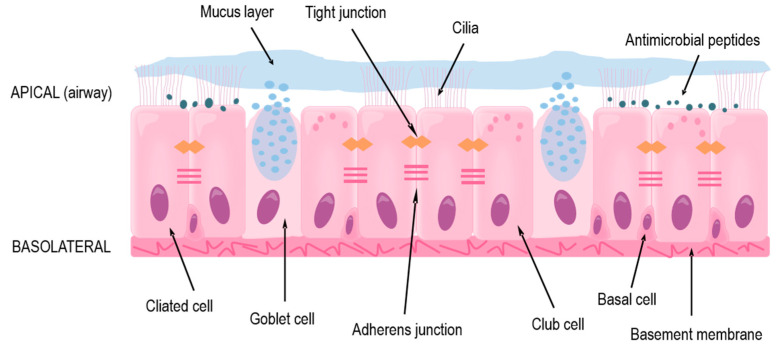
The structure and protective functions of the human airway epithelium in the lower respiratory tract.

The focus of this review is the lower conducting airways, or lower respiratory tract, which includes the trachea and the bronchi that branch out throughout each lung and end as terminal bronchioles just before the alveoli where gas exchange occurs. In the trachea and bronchi, the epithelium is pseudostratified with a clear apical-basolateral orientation and consists of ciliated cells, goblet cells, club cells, and the underlying basal cells (Figure 1). The ciliated cells together with goblet and club cells make up the mucociliary escalator on the apical side in which inhaled particles are trapped in secreted mucus and the beating cilia transport it upwards to the mouth where it is then swallowed or expectorated [10,11]. The basal cells comprise the stem-cell niche of the conducting airways by having the ability to differentiate into the other cell types mentioned above [12,13,14,15].

Under normal conditions, the mucociliary escalator ensures homeostasis by preventing any possible irritants or large pathogens from accessing the epithelial cells or underlying systemic circulation [11,16]. Additionally, airway epithelial cells produce various antimicrobial peptides (AMPs) which prevent microbes from colonizing the airways [17,18,19]. In asthma, both of these secretion-based protections are usually impaired, either due to endogenous genetic variations [20] or as an effect of an ongoing inflammation [9,21,22].

If inhaled agents escape the mucus or AMPs, through inherent properties or impairment of these defenses, they reach and can affect or infect the epithelial cells themselves [11,16]. Due to the tight barrier formed by the cells, many pathogens and allergens are prevented from reaching the circulation and accessing other cells and organs. The barrier is maintained by anchoring of the extracellular domains of proteins found near the cellular membrane and is organized into tight and adherens junctions, as well as desmosomes [23]. Tight junctions are located close to the apical side of the cells and are made up of transmembrane proteins occludin and members of the claudin and junctional adhesion molecule (JAM) families, which anchor to cytoplasmic proteins cingulin and members of the zonula occludens (ZO) family [23]. These proteins also interact with polarity proteins, which aid in the correct localization of the tight junctions; the tight junctions are then involved in maintaining the apical-basolateral polarity of the cells [24], which ensures the functionality of the epithelium. Beneath the tight junctions are adherens junctions, where transmembrane-spanning E-cadherin binds to intracellular p120-, β-, and α-catenin [25]. Several junctional proteins have been found to be disorganized or dysregulated in asthma [26,27], leading to an impaired barrier and dysfunction of the epithelium.

On the basolateral side of the airway epithelium is the basement membrane, consisting of extracellular matrix, to which the epithelial cells are strongly anchored through hemidesmosomes [28,29]. Farther below, there are airway smooth muscle cells, fibroblasts, and blood vessels, as well as cartilage rings around the trachea and first-generation bronchi [30]. These cells and structures, together with the epithelium, all contribute to the functionality of the airways and lungs through providing structure, contraction, and nutrients. There is also crosstalk between these underlying cells and the epithelium, mediated mainly through soluble factors [28,30,31].

On each side of the epithelial cells, and reaching between them, are both innate and adaptive immune cells. These cells include ILCs, DCs, mast cells, eosinophils, and T cells. In asthma, both the type and number of these cells are altered. Secreted factors produced by these cells, such as cytokines, proteases, and lipid mediators, affect the airway epithelium which in turn release alarmins and chemokines that affect the immune cells, creating a bridge between innate and adaptive immunity.

## 3. Genetic Associations with Asthma Linked to the Airway Epithelium

Several genomic screens and genome-wide association (GWA) studies have found genes and genetic loci associated with asthma that are expressed by the epithelium [20,32]. These findings highlight the importance of the airway epithelium in healthy individuals as well as in the pathology of asthma. One group of genes associated with asthma are related to the epithelial barrier function (see summary in ref. [33]); these include *PCDH1* (protocadherin-1), which is involved in cell adhesion and epithelial barrier formation [34] and *CDHR3* (cadherin-related family member 3), which is also involved in cell adhesion as well as epithelial polarity and is the receptor for rhinovirus (RV) C, where the risk variant could increase susceptibility to infection [35,36,37]. Additionally, *ORMDL3* (orosomucoid-like 3) has been linked to asthma in several populations [38,39,40], and its corresponding protein may be involved in cell adhesion and integrity; when increased, it has been shown to promote airway remodeling and hyperresponsiveness [41]. However, deletion of *ORMDL3* was also associated with increased airway hyperresponsiveness and remodeling [42], indicating that its mechanistic link to asthma risk is yet unknown. Other asthma-associated genes with possible roles in barrier function are *DPP10* (dipeptidyl peptidase 10) [43] and *GPRA* (G protein–coupled receptor for asthma susceptibility) [44].

Associations have also been found between variants of mucin-encoding genes (*MUC5AC* and *MUC5B*) and the risk for asthma [45,46], where the variants are predicted to cause increased mucin production. *CLCA1* (calcium-activated chloride channel regulator 1), which is involved in mucus secretion, has also been linked to asthma [47]. Lastly, several studies have linked polymorphisms in epithelial alarmins *TSLP* and *IL33* with the risk for asthma [36,39,46,48,49,50,51]. These findings indicate that genetic defects or variations within the airway epithelium can cause, drive, or worsen asthma, most likely driven through interactions with the environment. 

## 4. Impairment of the Airway Epithelial Barrier in Asthma

Compelling evidence indicates a range of abnormalities in various aspects of epithelial barrier function in asthma. Part of this impairment is a disruption of the airway epithelial layer, which may facilitate submucosal infiltration of inhaled substances and consequently their interaction with immune cells. In situ observations of the airway epithelium have revealed structural changes in asthmatic individuals, including patchy disruption of tight junctions, detachment of ciliated cells, and reduced expression of E-cadherin as well as other cell-cell adhesion molecules [26,27,52,53]. In line with these findings, functional studies of airway epithelial cells cultured at air-liquid interface (ALI) indicate increased permeability and sensitivity to environmental insults in cells from individuals with asthma compared with healthy controls [27,54,55]. Although the mechanisms contributing to loss of airway epithelial barrier function in asthma have not been fully elucidated, a combination of different extrinsic and intrinsic factors are likely to play a role.

Allergens, viral infections, and T2 inflammation are strongly associated with the pathogenesis of allergic asthma and are all considered to have detrimental effects on the barrier integrity of the airway epithelium. A number of in vitro studies have demonstrated the ability of various protease-containing allergens to disrupt the airway epithelial barrier, either directly or indirectly via activation of protease-activated receptor (PAR)-2, a proinflammatory innate immune receptor on epithelial cells [56]. The latter has been shown to cause loss of barrier integrity in house dust mite (HDM)-treated airway epithelial cells through a mechanism of epidermal growth factor receptor (EGFR) transactivation and subsequent E-cadherin destabilization [57]. The major allergen from HDM, *Dermatophagoides pteronyssinus* antigen P1 (Der p1), can also directly cleave the tight junction proteins occludin and ZO-1 [58,59]. In accordance, HDM extracts and Der p1 cause increased permeability and decreased transepithelial electrical resistance (TEER) in cultured airway epithelial cells [55,59]. Similar effects have been reported for other allergens, including the fungi *Alternaria alternata* (*Alternaria*) [53] and various pollen allergens [60,61,62]. 

In addition to promoting allergic sensitization, increased barrier permeability may also lower the threshold for epithelial damage and activation of a T2 response, which itself may affect barrier function, thus generating a positive feedback loop of increased epithelial permeability. Indeed, the two central T2 cytokines IL-4 and IL-13 have been found to induce barrier disruption by inhibiting the surface expression of ZO-1, occludin, E-cadherin, and β-catenin in bronchial epithelial cells [63,64]. Recently, ILC2s, which constitute an early source of T2 cytokines in asthma, were shown to induce increased epithelial barrier permeability and reduced expression of epithelial tight junction proteins via secretion of IL-13 in an ALI-coculture model of human bronchial epithelial cells (HBECs) and ILC2s [65]. IL-13 has also been found to decrease epithelial expression of claudin-18, the only known lung-specific tight junction protein [66]. Interestingly, lower expression of claudin-18 was identified in epithelial brushings from asthmatic individuals compared to healthy controls and loss of claudin-18 impaired epithelial barrier function both in vitro and in vivo [66].

Furthermore, infections with some respiratory viruses, such as influenza virus, can cause epithelial barrier dysfunction as a result of direct cytopathic effects. RV on the other hand cause little cell death, but have been shown to disrupt epithelial tight junctions by reducing occludin expression in a NADPH-oxidase-dependent manner, leading to increased airway epithelial permeability [67]. A recent study further demonstrated that RV infection caused loss of ZO-1 from tight junctions in ALI-cultured HBECs from asthmatic and healthy children, and that the effect was more pronounced and sustained in cells from children with asthma [68]. Moreover, infection with respiratory syncytial virus (RSV) has been found to cause adverse effects on airway epithelial junctional complexes through sustained activation of protein kinase D [69].

In addition to the impact of different environmental risk factors, the genetic background of the individual may also influence epithelial barrier properties. As described above, several susceptibility genes with potential implications in epithelial barrier function have been identified through GWA studies. Furthermore, epigenetic modifications serve as a secondary level of gene regulation that is likely to effect the translation of disease susceptibility into transformed airway epithelial biology. A more detailed discussion of epigenetic mechanisms in relation to airway epithelial barrier function will be given later in this review.

## 5. Airway Epithelial Responses to Inhaled Agents

It is now evident that the airway epithelium plays a key role in the initiation and orchestration of the immune response to various environmental factors. Inhaled agents such as aeroallergens, pollutants, and respiratory viruses are sensed by the airway epithelium via a diverse set of pattern recognition receptors (PRRs) like the toll-like receptors (TLRs), retinoic acid-inducible gene (RIG)-I-like receptors (RLRs), nucleotide-binding oligomerization domain (NOD)-like receptors (NLRs), C-type lectin receptors (CLRs), and PARs. Following activation of these receptors, airway epithelial cells release various inflammatory cytokines, chemokines, endogenous danger signals, and other mediators alarming and activating a variety of immune cells, importantly DCs and ILC2s. In the following sections, we will focus on some aspects of the interactions of airway epithelial cells with common aeroallergens and respiratory viruses and the effects on the airway epithelium in asthma.

### 5.1. Allergen-Airway Epithelial Interactions

As already highlighted, the airway epithelium does not simply act as a passive barrier hindering allergens from penetrating the mucosal surface, but is highly active in the recognition of allergens and initiation of innate immune responses that are critical for influencing the outcome of allergen inhalation. Allergens commonly involved in asthma development and exacerbation include dust mites, grass and tree pollen, animal dander, and fungi. These aeroallergens are complex mixtures of various constituents, including proteins with different structures and activities, which can interact with epithelial cells through diverse mechanisms. Importantly, repeated or sustained activation of epithelial PRRs, either by allergens themselves or by contaminating microbial pathogen-associated molecular patterns (PAMPs), has been proposed as one of the key steps in the modulation of DC-driven adaptive immune responses and the allergen sensitization process [70].

We have previously discussed the ability of certain protease-containing allergens to disrupt the airway epithelial barrier by acting on tight junctions. In addition, sensing of this protease activity by airway epithelial cells may also induce the release of various inflammatory mediators. For example, *Alternaria* has been shown to trigger protease-dependent PAR-2-mediated release of IL-6, CXCL8, and GM-CSF from HBECs in vitro [71], and similar effects have been found with cockroach proteases [72,73].

Furthermore, allergens can also activate airway epithelial cells via protease-independent mechanisms. Accordingly, HDM was reported to trigger protease-independent release of the DC-chemoattractant CCL20 via the interaction of HDM-derived β-glucan with the CLR dectin-1 in a bronchial epithelial cell line [74]. Additionally, the non-proteolytic HDM allergen Der p2, has been shown to induce airway epithelial release of CCL20, IL-6, CXCL8, GM-CSF, and MCP-1 via activation of NF-κB and MAPK pathways [75]. Surface expression of the intracellular adhesion molecule (ICAM)-1 was also upregulated on the same cells in response to Der p2, and this was associated with increased adhesion of monocytes to the epithelial cells [75]. Of note, ICAM-1 is also used as the receptor for cellular internalization by the major group RVs [76]. It has been suggested that at least part of the effects of Der p2 on airway epithelial cells could be due to TLR4 activation, since Der p2 shows high sequence homology with myeloid differentiation factor 2 (MD2), a TLR4 co-signaling molecule required for optimal TLR4 activation by LPS, and LPS is a known contaminating factor in extracts from HDM [77].

Thymic stromal lymphopoietin (TSLP), IL-33, and IL-25 are three epithelial-derived cytokines with critical roles in asthma pathogenesis as they are potent activators of DCs and ILC2s, which act upstream in the T2 immune response cascade. Exposure to aeroallergens in vitro has been shown to trigger epithelial release of all three cytokines [78,79,80,81]. Importantly, increased expression of TSLP, IL-33, and IL-25 was recently demonstrated in the airway epithelium of allergen-challenged individuals with mild atopic asthma and correlated with increased airway obstruction [82]. In addition, various allergens have been reported to trigger epithelial release of endogenous danger signals, such as ATP and uric acid [80,83], which may further influence DC and ILC2 behavior as well as amplifying the production of epithelial-derived T2-promoting cytokines [80].

Several in vitro studies have demonstrated synergy between allergens and different inflammatory mediators. For example, a study using HDM allergens showed that HDM acts synergistically with IL-4 and TGF-β, two mediators found to be increased in asthmatic airways [84,85], to trigger airway epithelial release of the Th2 cell chemoattractant CCL17 [86]. A further study found that IL-4 also increased *Alternaria*-induced release of TSLP, whereas the induction of TSLP was prevented by the type 1 (T1) cytokine interferon (IFN)-γ [78]. These findings suggest that the local microenvironment in the airways is likely to dictate the outcome of allergen-epithelial cell interactions, and may partly explain why allergens do not cause inflammation in healthy individuals, despite their capacity for direct activation of airway epithelial cells. Another important question is whether there is differential regulation of allergen-induced innate immune responses in asthmatic and healthy airway epithelium. Although few reports of altered epithelial innate immune responses to allergens in asthma are available to date, an in vitro study in HBECs demonstrated that cells from asthmatic individuals released more CCL20 compared with cells from healthy controls in response to stimulation with the HDM allergen Der p1 [87], indicating that such dysregulation may exist.

### 5.2. Virus-Airway Epithelial Interactions

In healthy individuals, upper respiratory tract viral infections are usually self-limiting and manifest as a common cold with relatively mild symptoms. In individuals with asthma, however, respiratory viruses, particularly RV, are able to subvert host immune defense systems and act as major triggers of exacerbations in both children and adults [88,89,90]. These acute, disease-worsening events impair quality of life, are a major cause of hospitalization, and can, in their most severe form, be fatal [91]. In addition to the causative role of viruses in asthma exacerbations, there is considerable evidence that virus-induced wheezing illnesses early in life are a significant risk factor for later asthma development, especially in genetically susceptible children [92,93,94]. Again, this association is particularly strong with RV [92,93,94,95,96], but RSV has also been suggested to be a risk factor [97,98].

Although important progress has been made over the past decade, the precise pathogenic mechanisms by which respiratory viruses may drive asthma inception and exacerbations are not completely understood. Bronchial epithelial cells are the primary targets of respiratory viruses and the main site of viral replication in the lower airways [99,100]. Hence, dysregulated epithelial production of mediators that influence the immune response has been suggested as one explanation to why respiratory infections trigger asthmatic and allergic reactions in susceptible individuals. Even though RSV, influenza virus, and some additional respiratory viruses have been detected in airway samples from asthma patients with exacerbating disease, RV infections are by far the most frequent cause of viral-induced asthma exacerbations, accounting for up to 70–80% of all cases [101]. As a consequence, experimental studies on epithelial responses to RV infection in asthma currently dominate the research field.

Early host recognition of respiratory viruses by the airway epithelium mainly occurs through a number of PRRs that sense viral RNA. Replication of single-stranded RNA viruses such as RV leads to the production of double-stranded RNA (dsRNA), which is recognized as a potent stimulus for antiviral innate immune responses [102]. Airway epithelial cells constitutively express TLR3, which in a coordinated manner with the IFN-inducible RLRs melanoma differentiation-associated gene (MDA)-5 and RIG-I, interacts with dsRNA, leading to activation of downstream signaling pathways involving NF-κB and IRF3/IRF7 [102,103]. Ultimately, this results in the production of type I (IFN-α/β) and III (IFN-λ1, 2, and 3) IFNs [104]. In addition, a wide range of proinflammatory cytokines (IL-1β, IL-6), chemokines (CXCL8, CXCL5, CXCL10, CCL5/RANTES), and growth factors (G-CSF, GM-CSF) are produced, which can contribute to the activation and recruitment of various immune cells to the airways [104].

Epithelial generation of IFNs is essential for effective antiviral responses and viral clearance. IFNs are able to induce hundreds of IFN-stimulated genes, which cooperate to limit viral replication and invasion by a number of mechanisms [105]. Despite some controversial reports, there is evidence that RV-induced epithelial production of IFNs is reduced in some individuals with asthma, providing one plausible explanation to the increased susceptibility to viral infections in at least a subgroup of asthmatic individuals. Wark et al. were the first to demonstrate impaired IFN production in the asthmatic epithelium. In their study they found that HBECs from subjects with asthma exhibited increased RV replication in vitro compared with healthy individuals, and that this was reflected by delayed and deficient IFN-β induction [106]. Contoli et al. later made similar observations of deficient IFN-λ induction in RV-infected HBECs from atopic individuals with asthma [107]. By using a human experimental model of RV exacerbation, the authors further showed that exacerbation severity was inversely proportional to IFN-λ generation. Though there have been contradictory results [108,109,110], these initial findings have since been confirmed in several reports [111,112,113,114,115,116], and different factors have been proposed to negatively regulate RV-induced epithelial IFN production, including suppressor of cytokine signaling (SOCS)1, the T2 cytokines IL-4 and IL-13, HDM, and oxidative stress [117,118,119,120]. Despite extensive research, however, mechanistic insight into the impaired IFN responses in asthmatic individuals is still lacking and further studies are warranted.

Viral infections are classically associated with the induction of a T1 immune response. Yet, emerging evidence suggests the involvement of the T2-promoting cytokines IL-25, IL-33, and TSLP in the response to respiratory viruses and associated exacerbations in asthma. By using a human experimental model of RV exacerbation, Jackson et al. found that subjects with asthma had increased levels of IL-33 and that this correlated with the T2 cytokines IL-5 and IL-13 in the airway lining fluid as well as exacerbation severity after virus inoculation [121]. They also showed that supernatants from RV-infected HBECs triggered IL-33-dependent induction of IL-4, IL-5, and IL-13 in human T cells and ILC2s in vitro. In a similar model, Beale et al. showed that IL-25 was induced by experimental RV infection, and that IL-25 expression both at baseline and during infection was higher in asthmatic individuals [122]. They further demonstrated in vitro that RV-infected HBECs from asthmatic subjects had greater IL-25 induction compared with cells from healthy individuals. Moreover, several studies have found overproduction of TSLP in response to dsRNA or RSV infection ex vivo in HBECs from asthmatic individuals compared with healthy controls [112,123,124]. Taken together, these data suggest that airway epithelial cells from asthmatic individuals may have an increased capacity for IL-33, IL-25, and TSLP production in response to virus infections, and that these cytokines may be important mediators in exaggerated T2 inflammatory responses in viral-induced asthma exacerbations. The biological functions of IL-33, IL-25, and TSLP, as well as their proposed roles in T2 immunity and asthma, will be further discussed below.

## 6. Epithelial-Derived Cytokines as Master Regulators of T2 Immunity

**Figure 2:**
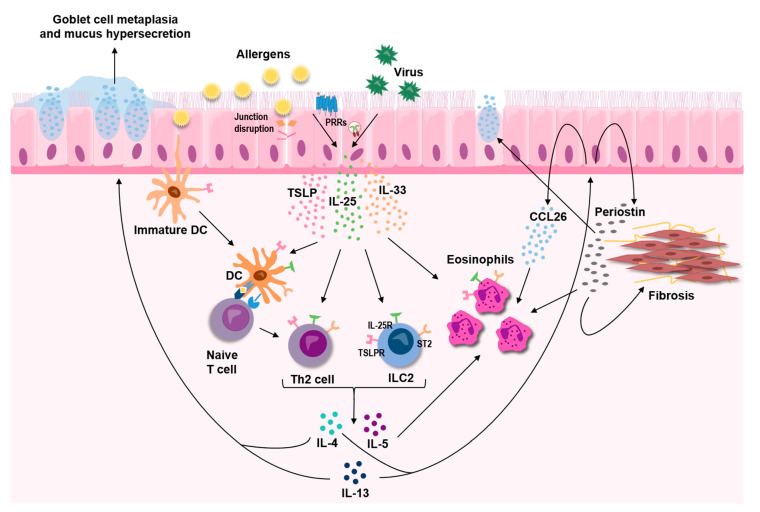
The epithelial-derived cytokines TSLP, IL-33, and IL-25 are released in response to various insults, including allergens and respiratory viruses, and act as key upstream drivers of type 2 inflammation in the airways through effects on both innate and adaptive immune cells.

Overwhelming evidence supports a central role for the three epithelial-derived cytokines, TSLP, IL-33, and IL-25, in asthma. These three cytokines, commonly referred to as alarmins, act as master regulators that mediate both innate and adaptive immune responses, leading to sustained T2-skewed inflammation (Figure 2). Although distinct in their mode of action, crosstalk within this triad of alarmins is likely to exist and is underpinned by the findings that some of the triggers for release are shared by all three cytokines. In light of the strong indication that they act as upstream drivers of T2-mediated disease, TSLP, IL-33, and IL-25 have attracted a lot of interest as potential therapeutic targets in asthma, and monoclonal antibodies targeting TSLP and IL-33 are currently under clinical evaluation.

### 6.1. TSLP

TSLP is a member of the IL-2 cytokine family and considered a pivotal upstream cytokine driving a pronounced T2 immune response [125,126,127]. The airway epithelium is a major source of TSLP under both homeostatic and inflammatory conditions [128,129,130]. A range of stimuli involved in asthma pathogenesis, including respiratory viruses, proinflammatory (TNF-α and IL-1β) and T2 (IL-4 and IL-13) cytokines, and proteolytic allergens have been shown to cause increased expression and release of TSLP from airway epithelial cells through activation of different PRRs and cytokine receptors, supporting its function as an alarmin signaling a compromised airway epithelium [78,123,126,131,132,133,134].

TSLP binds to a heterodimeric receptor complex composed of the TSLP receptor (TSLPR) and the IL-7Rα chain. The broad effect of TSLP on the immune response in the airways is reflected by the various cell types that express the TSLPR, including many cells of the hematopoietic system, but also structural cells like airway smooth muscle cells [135,136]. The role of TSLP in asthma and allergic inflammation has been extensively investigated. Both in vitro and in vivo studies have demonstrated a strong link between TSLP expression and the production of IL-4, IL-5, and IL-13, which are central in the development of a T2 phenotype in asthma [125,133,137,138,139,140]. It is believed that a major T2 promoting effect of TSLP is its ability to induce OX40 ligand on DCs, priming them to drive differentiation of naive CD4+ T cells into functional Th2 cells, which produce IL-4, IL-5, and IL-13 [125,139,140]. In addition, TSLP can interact directly with other cells of the immune system, such as mast cells, eosinophils, Th2 cells, and ILC2s to promote a T2-biased inflammatory response [126,133,138,141,142,143].

As previously mentioned, several single-nucleotide polymorphisms (SNPs) in the TSLP gene associated with increased asthma risk have been identified through GWA studies, indicating a role of TSLP in asthma pathogenesis [39,48,144]. In support of these findings, a number of studies have shown that TSLP expression is elevated in the airway epithelium and bronchoalveolar lavage (BAL) fluid of individuals with asthma and that it correlates with disease severity and loss of lung function [128,129,130]. Furthermore, clinical trials have now provided strong evidence for a central role of TSLP as an important modulator in asthma. A humanized monoclonal anti-TSLP antibody, tezepelumab, which blocks the interaction of TSLP with TSLPR recently completed a phase 2 clinical trial for uncontrolled, severe asthma where tezepelumab-treated individuals displayed significant reduction in asthma exacerbation rate together with improved lung function and asthma control [145]. In addition, an earlier clinical trial demonstrated that anti-TSLP reduced bronchoconstriction and airway inflammation in mild asthmatic individuals before and after allergen challenge [146].

### 6.2. IL-33

IL-33 is a member of the IL-1 superfamily and has been forwarded as a multifactorial alarmin cytokine with critical roles in T2 immunity and asthma pathophysiology [147]. Airway epithelial cells are the primary cell type in the human airways that express IL-33 under basal conditions, where it is predominantly localized to the nucleus in a full-length precursor form [148,149]. Cellular release of immunologically active full-length IL-33 (IL-33_FL_) occurs rapidly following epithelial injury or exposure to environmental stressors such as airborne allergens and viruses [80,81,121]. Although IL-33 has primarily been considered to be passively released due to cell necrosis, findings also indicate active mechanisms of IL-33 secretion in response to allergens, mediated via purinergic receptor-dependent signaling or dual oxidase 1 (DUOX1)-dependent activation of EGFR-signaling [80,81].

Similar to other IL-1 family cytokines, the activity of IL-33 is regulated by both its cellular localization and by proteolytic cleavage. Recent studies have shed new light on how IL-33 activity can be regulated by direct sensing of proteolytic activities, as well as oxidative changes. Cayrol et al. demonstrated that various allergens with protease activity, including HDM, *Alternaria*, *Aspergillus fumigatus* and pollens, can induce IL-33_FL_ release and subsequent cleavage in a central sensor domain into a shorter form with considerably enhanced bioactivity that potently stimulates ILC2s [150]. In another study, Scott et al. demonstrated that IL-33 activity can be enhanced by proteolytic mechanisms involving allergen proteases as well as endogenous proteases from damaged airway epithelial cells [151]. In addition, they showed that allergen proteases degraded mature oxidized forms of released IL-33, suggesting a regulatory mechanism for rapid inactivation of IL-33 in an oxidative milieu, such as during tissue injury.

IL-33 binds to a heterodimeric receptor formed by IL-1 receptor-like 1 (IL1RL1, also known as ST2) and the IL-1 receptor accessory protein, which leads to activation of NF-kB and MAPK signaling pathways [147,152]. In addition to membrane-bound ST2, there is also a soluble form (sST2), which can act as a decoy receptor to sequester free IL-33, preventing IL-33/ST2 signaling [152]. The most established function of IL-33 is activation of ST2 expressing immune cells involved in T2 immunity, such as ILC2s, Th2 cells, mast cells, eosinophils, basophils, and DCs [153,154,155,156,157,158,159]. The functional role of IL-33 in T2 immunity-associated allergic responses and asthma has been extensively investigated in vivo and numerous studies have shown that inhibition of IL-33/ST2 signaling attenuates T2 inflammation in murine models of allergic asthma [155,157,160,161,162].

Significant associations between genetic variants of *IL33* and *IL1RL1* and human asthma have consistently been identified in several genetic studies, suggesting that the IL-33/ST2-axis is likely to play a role in the disease [39,144,163]. In support of this, IL-33 has been shown to be upregulated in the airway epithelium and BAL fluid from individuals with moderate to severe asthma, and release of IL-33 is increased during experimental RV-induced asthma exacerbation [121,149]. Clinical phase 2 trials with monoclonal antibodies targeting either IL-33 or ST2 are currently ongoing and will evaluate the potential of IL-33 as a therapeutic target in asthma.

### 6.3. IL-25

IL-25, also known as IL-17E, is a member of the IL-17 cytokine family, consisting of six structurally related but functionally distinct proteins [164]. Whereas other IL-17 cytokine members such as IL-17A and IL-17F seem to have important roles in neutrophilic inflammation, proinflammatory cytokine induction, and T1 immunity, IL-25 is unique in that it promotes T2 immune responses, including eosinophilic inflammation and overproduction of IL-4, IL-5, and IL-13 [165]. A specialized group of epithelial cells called solitary chemosensory cells were recently identified to be the main epithelial source of IL-25 in the upper airways [166]. Airway epithelial cells have been demonstrated to contain preformed IL-25, which is stored or sequestered in the cytoplasm [79]. When exposed to protease-containing allergens such as HDM, epithelial cells rapidly release IL-25, implying a role in allergic disease [79]. Other proteases, such as papain and trypsin, or breakdown of cell-cell adhesion molecules may also trigger epithelial release of IL-25 [79,167]. The exact mechanisms of IL-25 release and regulation, however, have yet to be defined.

IL-25 binds to a heterodimeric receptor, IL-17RA/IL-17RB (IL-25R), which is expressed on several cell types such as ILC2s, activated memory Th2 cells, TSLP-activated DCs, mast cells, eosinophils, and endothelial cells [164,168,169,170]. Thus, IL-25 is able to mediate both innate and adaptive immune responses to induce a sustained T2-biased mucosal inflammation. For example, IL-25 may amplify Th2-cell dependent pathways leading to enhanced allergic inflammation [171]. Although IL-33 seems to be superior in driving the development and activation of ILC2s, IL-25 also functions as an ILC2-inducing cytokine [159].

Studies in both mice and humans suggest a role for IL-25 in asthma. Different experimental murine models have shown that allergic inflammation can be attenuated by blocking IL-25 signaling [122,167,172]. In a study by Cheng et al., the role of IL-25 in the lower airways was investigated in steroid naive, newly diagnosed asthmatic individuals and healthy control subjects [173]. By analyzing bronchial brushings and biopsies, BAL, sputum, and blood, the authors could reveal an “IL-25-high” subgroup among asthmatic individuals who exhibited increased airway epithelial expression of IL-25 associated with severe airway eosinophilia, marked subepithelial fibrosis, higher expression of *MUC5AC* and elevated IgE levels. Further, plasma IL-25 levels correlated with epithelial IL-25 expression, suggesting that IL-25 may have potential as a systemic biomarker for stratifying patients for treatment. As previously described, a role for IL-25 in viral-induced asthma exacerbations has also been indicated [122]. Although the collected data supports an association between IL-25 and asthma, clinical trials using anti-IL-25 antibodies, as for TSLP and IL-33, have not yet been conducted.

## 7. The Effect of T2 Inflammation on the Airway Epithelium

Airway epithelial cells are able to respond to many cytokines, both pro- and anti-inflammatory, including the key T2 cytokines IL-4, IL-5, and IL-13 [174,175,176]. Several studies by Woodruff and colleagues have demonstrated the ability of the airway epithelium to respond with unique gene and protein signatures in response to T2 cytokines, and that these signatures could be used as biomarkers for T2 asthma [177,178,179]. One of the proteins from this T2 signature is periostin, which is increased in epithelial cells in response to IL-13 +/− IL-4 [177,180,181], and which may have potential as a systemic biomarker of eosinophilic airway inflammation [182,183]. Periostin, a matricellular protein, has been implicated in processes related to airway remodeling such as cell proliferation, collagen production and epithelial-to-mesenchymal transition [184], as well as subepithelial fibrosis [185], mainly driven by TGF-β activation. Periostin could also increase the expression of mucin genes in airway epithelial cells [186], as well as act as a binding partner for eosinophils and thereby promote their migration [187], both processes involved in T2 airway inflammation and asthma (Figure 2).

A widely studied effect from T2 cytokines on the epithelium is the induction of mucus production [11,188], an important clinical feature in asthma (Figure 2). The effect is mediated by altered expression and secretion of the mucins MUC5AC and MUC5B, which have different viscoelastic properties and are therefore believed to have different roles in the human airways. MUC5AC is commonly found to be increased in asthmatic individuals and in airway epithelia exposed to IL-13, whereas there are conflicting results around levels of MUC5B, meaning that the ratio between the two mucins may be altered in asthma, leading to altered properties of the mucin gel layer [189,190]. Linked to increased mucus production are SPDEF (SAM pointed domain-containing Ets transcription factor) and CLCA1, both also induced by T2 cytokines in the airway epithelium and the latter, as mentioned previously, is also linked genetically to asthma and part of the epithelial T2 signature introduced above [47,177,191,192]. Out of these, SPDEF, a transcription factor, appears to be most critical for goblet cell hyperplasia and increased mucus release in human [191,193], which when silenced or knocked out abolishes goblet cell differentiation and mucus release, by decreasing both CLCA1 and MUC5AC [192,194]. How CLCA1 modulates mucus production and secretion is, however, not yet fully elucidated and points towards possible differences between mice and man [195,196], but there are indications that CLCA1 could activate MAPK13 which in turn increases MUC5AC expression [197] whereas blocking chloride channels by niflumic acid lowers MUC5AC expression [198].

The increase in mucus production can lead to mucus plugging and impaired mucociliary clearance, the latter possibly due to IL-13-induced tethering of the mucus gel to the epithelial cells rather than impaired ciliary function [199]. A recent single-cell sequencing study identified a novel mucous ciliated cell state in asthma, induced by T2 cytokines, which could contribute to mucous cell metaplasia [200]. Mechanistic studies are being employed to further understand this effect and thus aid in how to possibly modulate mucus hypersecretion in asthma. Studies have highlighted the importance of autophagy in IL-13-induced mucus production [201], as well as the involvement of NOTCH3 signaling [202], both providing possible pathways for therapeutic intervention. The airway epithelium also responds to T2 cytokines by secreting proteins that could aid in counteracting impaired mucociliary clearance. An example of this being gelsolin, which has been found to be increased in epithelial cell cultures treated with IL-4, where it may improve fluidity of the airway surface liquid through the breakdown of released filamentous actin [203].

Another important clinical feature in asthma is increased nitric oxide, a gas involved in airway reactivity and inflammation, which is detectable in exhaled breath and used as a biomarker for T2 asthma [204,205,206,207]. Higher levels of nitric oxide are usually linked to increased levels of inducible nitric oxide synthase (NOS2) in the airway epithelium, which can be normalized through the administration of corticosteroids [208,209,210]. Several cytokines can increase the levels of NOS2 on both the gene and protein level [208,210], though the effect is suggested to be stronger from T2 cytokines including IL-4 and IL-13 [181,211].

**Figure 3:**
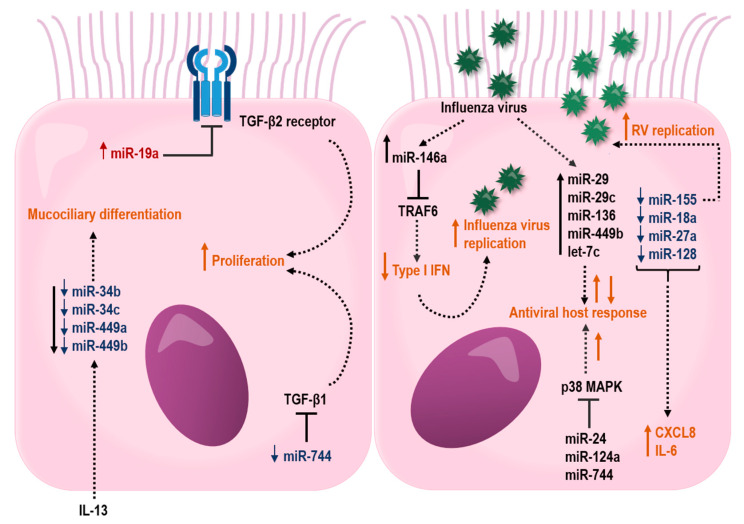
Proposed effects of miRNAs in the airway epithelium that may influence barrier functions and host response to respiratory virus infections. miRNAs that are up- or downregulated in bronchial epithelial cells from asthmatic individuals are depicted in red color with upward arrow and blue color with downward arrow, respectively. Black lines ending with a perpendicular line indicate an inhibitory effect. Dotted lines illustrate regulated miRNAs or processes (orange color), where up- and downward arrows indicate stimulation and suppression, respectively.

A key inflammatory cell in T2 asthma is the eosinophil, of which there are increased numbers both locally and systemically in individuals with asthma. Eosinophils are recruited by eotaxins, a group of chemokines with three members in humans, comprised of CCL11 (eotaxin-1), CCL24 (eotaxin-2), and CCL26 (eotaxin-3). Of these, CCL26 has been shown to be the strongest inducer of eosinophil migration in asthmatic individuals [212]. Several studies have shown that this chemokine is strongly induced by IL-13 in airway epithelial cells (Figure 3) [181,207,213,214] and further that it is elevated in serum and bronchial biopsies from asthmatic individuals [183,207] and correlates with sputum eosinophil counts [213]. Recently, interest has grown when it comes to the involvement of extracellular vesicles, or exosomes, in asthma pathology and their potential as biomarkers. Airway epithelial cells have been shown to secrete extracellular vesicles that could be involved in cell-cell communication both during homeostasis and disease. These epithelium-derived vesicles tend to be coated with mucins, and the number of vesicles increase upon stimulation with T2 cytokines [179,215,216,217,218]. Additionally, in T2 conditions, epithelium-derived exosomes can induce chemotaxis of macrophages [206] and their cargo, such as proteins and microRNA (miRNA)s, are altered [181,219,220], all possibly contributing to pathological pathways observed in asthma.

## 8. Epigenetic Regulation of the Airway Epithelium in Asthma

The common feature of epigenetic mechanisms is that they regulate gene expression without affecting the nucleotide sequence of the genomic DNA [221]. The classical epigenetic modifications are DNA methylation and histone modifications, both of which are highly relevant to asthma pathophysiology. A recent study by Stefanowicz et al. showed six histone modifiers to be differently expressed in airway epithelial cells derived from individuals with asthma as compared to healthy individuals [222,223]. In addition to human studies, beneficial effects by targeting histone deacetylases have been carried out in animal models of asthma (can be reviewed in [224]). However, DNA methylation is probably the most studied epigenetic modification in general, but also in asthma. Indeed, a recent study demonstrated that SNPs identified by GWA studies affect asthma risk through DNA methylation and expression of *cis*-genes in airway epithelium [225]. In another study, bronchial mucous tissues obtained from atopic or non-atopic individuals with asthma revealed similar DNA methylation levels as in healthy control. Importantly, a set of loci was identified with significant differences in DNA methylation between the asthma groups [226]. Several studies have demonstrated DNA methylation changes in asthma genes, induced by environmental factors [227,228,229]. RV infection was shown to induce DNA methylation changes in nasal epithelial cells derived from both children and adults with asthma. Furthermore, diesel exhaust particle exposure and allergen challenge induced DNA methylation patterns in human airway epithelial cells 48 h post exposure. In addition to DNA methylation changes, airway epithelial gene expression can be modulated by miRNAs. Thus, miRNAs have shown to be differentially expressed in bronchial epithelial cells following tobacco smoke exposure, diesel exhaust particle exposure, and virus exposure (reviewed in [230]). These data highlight the role of environmental exposures in epigenetic regulation of gene expression in airway epithelium in asthma.

### 8.1. Epigenetic Regulation of the Airway Epithelium through miRNAs

Although the classical epigenetic modifications mentioned above are the most studied mechanisms to date, noncoding RNAs such as miRNAs are involved in the epigenetic regulation of epithelial gene expression. miRNAs are gene-regulatory small noncoding RNAs that bind to target mRNAs leading to mRNA degradation and in some cases translational repression [231,232,233]. miRNAs are crucial in most biological and pathological processes, including immune responses, cell proliferation, cell differentiation, and apoptosis. To date there are a limited number of studies examining miRNA expression in the airway epithelium of asthmatic individuals compared to healthy individuals. However, one of the first studies using bronchial brushing samples of airway epithelial cells from asthmatic individuals, cultured at ALI, demonstrated higher expression of let-7f, miR-181c, -487b and lower expression of miR-203 compared with healthy control samples. Network analysis has suggested the aquaporin gene *AQP4* as a target gene of miR-203 and this gene was also shown to be highly increased in cells from asthma patients [234]. However, recent studies have identified Abelson tyrosine kinase (Abl) as a target for miR-203, which may have functional impact on epithelial cell proliferation, adhesion, growth, and development [235]. Therefore, a reduced level of this miRNA in airway epithelium in asthma may lead to cell proliferation and goblet cell hyperplasia [234].

In another study, bronchial brushings from asthma patients have shown a reduced expression of miR-181b, which was inversely correlated with sputum eosinophilia. Interestingly, overexpression of miR-181b reduced levels of IL-13 induced secretion of IL-1B and the eosinophil chemoattractant, eotaxin-1 (CCL11) by targeting SSP1 in a bronchial epithelial cell line. Furthermore, dexamethasone restored IL-13-induced miR-181b downregulation and inhaled corticosteroid treatment increased miR-181b in plasma from asthma patients. This study highlights a possible role for miR-181b as a biomarker for eosinophilic asthma [236]. In addition, the miR-34/449 family (miR-34b, miR-34c, miR-449a, and miR-449b) were shown to be suppressed in bronchial brushings from individuals with asthma, which was associated with an increased IL-13 expression (Figure 3). Interestingly, IL-13-induced inhibition of miR-34/449 family members resulted in an altered mucociliary differentiation towards a reduced number of ciliated cells and increased number of mucous cells, suggesting a role for this miRNA family in asthma pathogenesis [237].

A recent study has suggested that reduced levels of miR-146a in HBECs from asthma patients may contribute to neutrophilic asthma. However, reduced levels of miR-146a were found irrespective of asthma phenotype, but the neutrophil chemoattractant CXCL8 and CXCL1 were only increased in the neutrophilic asthma phenotype [238]. Both stimulated and unstimulated HBECs transfected with miR-146a mimics revealed reduced levels of both CXCL8 and CXCL1 mRNA. Even though the exact target for miR-146a was not identified, this study suggests that reduced levels of miR-146a may contribute to the development of a neutrophilic asthma phenotype [238].

### 8.2. miRNAs and the Airway Epithelial Barrier in Asthma

Little is known about how miRNAs influence the epithelial barrier function in asthma. However, studies have suggested that the impact of miRNAs on the airway epithelium may differ with severity of disease. Notably, several differentially expressed miRNAs in airway epithelial cells target genes involved in airway epithelial barrier functions (Figure 3). In severe asthma, miR-19, -221, and -744 were shown to be differently expressed in HBECs, specifically from individuals with an eosinophilic allergic asthma [239,240,241]. miR-19a was shown to enhance proliferation of HBECs, specifically in severe asthmatics by targeting the TGF-β2 receptor in HBECs [240]. In contrast to miR-19 and -221, the expression miR-744 was reduced in HBECs from severe asthma. This miRNA inhibits proliferation of HBECs by regulating the Smad3 pathway via targeting TGF-β1, a major proinflammatory mediator involved in fibrotic tissue remodeling within the asthmatic lung, highlighting a possible role for this miRNA in asthma pathogenesis [241].

### 8.3. miRNAs and Airway Epithelial Cell Responses to Virus Infection

Common viruses that affect the respiratory system are human RVs, RSV, and influenza viruses. As previously described, these viruses are known to cause illness and exacerbations in individuals with asthma. One of the most studied miRNAs is miR-155, which, in addition to regulating T2 inflammation in animal models of asthma [242,243], has been shown to be involved in RV replication in HBECs [244]. Inhibition of miR-155 in HBECs resulted in an increased viral replication of RV-1B [244]. An additional study demonstrated decreased expression of miR-18a, -27a, -128 and -155 in HBECs derived from individuals with asthma, and further knockdown of these miRNAs led to increased expression of the proinflammatory cytokines IL-6 and CXCL8. Indeed, IL-6 and CXCL8 are known to be increased in HBECs from asthmatics both under baseline conditions and after stimulation, suggesting a regulatory role for miRNAs in the control of IL-6 and CXCL8 expression in asthma [245].

In addition, miRNAs have also been shown to take part in essential pro-viral host factors through their regulation of the downstream p38 MAPK kinases, MK2 and Myc. Thus, miR-24, -124a, and -744 were shown to have antiviral effects on influenza A virus in the human lung epithelial cell line A549, whereas miR-124a and -744 had antiviral effects in RSV infection. These antiviral effects were through the suppression of the p38 MAPK pathway [246]. Influenza A viruses also increase the expression of miR-29, -29c, -136, 449b, and let-7c in A549 cells [247,248,249,250,251,252]. All of these miRNAs affect the influenza A viral response by targeting genes involved in antiviral host defense. In addition, miR-146a is induced in influenza virus A infection and downregulation of miR-146a was shown to inhibit influenza A virus replication by enhancing IFN type 1 responses by directly targeting the tumor necrosis factor receptor association factor 6 (TRAF6) [253].

Together, these data suggest a role for miRNA regulation of immune responses to respiratory viruses (Figure 3), and it is tempting to speculate that the miRNAs that affect virus replication therefore play a pivotal role in virus-induced exacerbations in asthma.

## 9. Conclusions

Today it is evident that the airway epithelium is not merely a passive barrier, but an essential part of the local immune response in the airways, bridging innate and adaptive immunity against various environmental insults. Overwhelming evidence indicates that the airway epithelium is dysfunctional in asthma, and plays a critical role in the development, progression, and exacerbation of the disease. This impairment includes both structural and immunological components, which collectively may influence the outcome of environmental challenges and contribute to asthma pathology. A role of the airway epithelium in asthma is further supported by findings from GWA studies, where many genes associated with asthma development are expressed in the airway epithelium.

Furthermore, epigenetic regulatory mechanisms may also contribute to abnormalities in asthma. miRNAs have recently been recognized as important modulators of airway epithelial functions. Although several studies have described differential expression of miRNAs in the asthmatic airway epithelium, the functional consequences of such dysregulation have not been investigated to the same extent. Hence, more mechanistic studies delineating the role of miRNAs in various aspects of airway epithelial dysfunction, including barrier impairment and deficient antiviral responses, are warranted, and could potentially pave the way for new treatment strategies in asthma.

About half of all individuals with asthma exhibit active T2 inflammation. It is now recognized that factors beyond IgE-mediated allergen sensitization, such as respiratory viruses, can trigger T2 immune responses. In addition, various allergens, including HDM, have the capacity to stimulate T2-skewed innate immune responses through epithelial PRR activation. The airway epithelium is a major source of TSLP, IL-33, and IL-25, which are rapidly released in response to both viruses and allergens. These alarmin cytokines activate various cells of both the innate and adaptive immune system, importantly DCs and ILC2s, thus acting as key upstream drivers of the T2 inflammatory cascade in asthma. This notion has led to an immense interest in targeting these epithelial-derived cytokines as a potential treatment strategy in asthma, and recent clinical trials blocking TSLP have shown promising results.

When activated by the alarmins, immune cells secrete T2 cytokines, which further affects the airway epithelium. This includes increased production of mucus and immune cell recruiting chemokines, as well as effects on the epithelial barrier such as remodeling and fibrosis. These changes thus lead to sustained inflammation and a persisting disease. Additionally, the epithelium is a potential source of both local and systemic biomarkers in the form of for example proteins or miRNAs, as the large surface area of the epithelium would enable production of high enough levels to be detected.

Taken together, it is clear that the airway epithelium is a central player in asthma, and further insight into the regulatory mechanisms underlying airway epithelial dysfunction could identify novel targets for future asthma intervention.

## Abbreviations

AMP	Antimicrobial peptide
DC	Dendritic cell
ILC2	Type 2 innate lymphoid cell
GWA	Genome-wide association
ALI	Air-liquid interface
T2	Type 2
PAR-2	Protease-activated receptor 2
HDM	House dust mite
Th2	T helper 2
EGFR	Epidermal growth factor receptor
RV	Rhinovirus
RSV	Respiratory syncytial virus
PRR	Pattern recognition receptors
TLR	Toll-like receptor
HBEC	Human bronchial epithelial cell
TSLP	Thymic stromal lymphopoietin
IFN	Interferon
dsRNA	Double-stranded RNA
SNP	Single nucleotide polymorphism
BAL	Bronchoalveolar lavage
miRNA	microRNA
